# *Sodalis glossinidius* presence in wild tsetse is only associated with presence of trypanosomes in complex interactions with other tsetse-specific factors

**DOI:** 10.1186/s12866-018-1285-6

**Published:** 2018-11-23

**Authors:** Manun Channumsin, Marc Ciosi, Dan Masiga, C Michael R Turner, Barbara K Mable

**Affiliations:** 1 Institute of Biodiversity, Animal Health and Comparative Medicine (BAHCM), Graham Kerr Building, University of Glasgow, University Place, Glasgow, G12 8QQ UK; 2 Faculty of Veterinary Medicine, Rajamangala University of Technology Tawan-Ok, Chonburi, 20110 Thailand; 3 International Centre of Insect Physiology and Ecology (ICIPE), P.O. Box 30772, Nairobi, 00100 Kenya; 4 Institute of Infection, Immunity and Inflammation, Sir Graeme Davis Building, University of Glasgow, University Place, Glasgow, G12 0PT UK

**Keywords:** *Trypanosoma vivax*, *Trypanosoma brucei*, *Trypanosoma congolense*, *Glossina austeni*, *Glossina brevipalpis*, *Glossina longipennis*, *Glossina pallidipes*, Secondary endosymbionts, Vector-pathogen interactions, Kenya

## Abstract

**Background:**

Susceptibility of tsetse flies (*Glossina* spp.) to trypanosomes of both humans and animals has been associated with the presence of the endosymbiont *Sodalis glossinidius.* However, intrinsic biological characteristics of the flies and environmental factors can influence the presence of both *S. glossinidius* and the parasites. It thus remains unclear whether it is the *S. glossinidius* or other attributes of the flies that explains the apparent association. The objective of this study was to test whether the presence of *Trypanosoma vivax, T. congolense* and *T. brucei* are related to the presence of *S. glossinidius* in tsetse flies when other factors are accounted for: geographic location, species of *Glossina*, sex or age of the host flies.

**Results:**

Flies (*n* = 1090) were trapped from four sites in the Shimba Hills and Nguruman regions in Kenya. Sex and species of tsetse (*G. austeni, G. brevipalpis*, *G. longipennis* and *G. pallidipes*) were determined based on external morphological characters and age was estimated by a wing fray score method. The presence of trypanosomes and *S. glossinidius* was detected using PCR targeting the internal transcribed spacer region 1 and the haemolysin gene, respectively. Sequencing was used to confirm species identification. Generalised Linear Models (GLMs) and Multiple Correspondence Analysis (MCA) were applied to investigate multivariable associations. The overall prevalence of trypanosomes was 42.1%, but GLMs revealed complex patterns of associations: the presence of *S. glossinidius* was associated with trypanosome presence but only in interactions with other factors and only in some species of trypanosomes. The strongest association was found for *T. congolense,* and no association was found for *T. vivax*. The MCA also suggested only a weak association between the presence of trypanosomes and *S. glossinidius*. Trypanosome-positive status showed strong associations with sex and age while *S. glossinidius-*positive status showed a strong association with geographic location and species of fly.

**Conclusions:**

We suggest that previous conclusions about the presence of endosymbionts increasing probability of trypanosome presence in tsetse flies may have been confounded by other factors, such as community composition of the tsetse flies and the specific trypanosomes found in different regions.

**Electronic supplementary material:**

The online version of this article (10.1186/s12866-018-1285-6) contains supplementary material, which is available to authorized users.

## Background

In sub-Saharan Africa, Animal African Trypanosomiasis (AAT) is caused primarily by three species of trypanosomes that are transmitted by tsetse fly vectors: *Trypanosoma vivax*, *T. congolense* and *T. brucei*. The disease causes dramatic losses of farm animal production [1] and leads to economic losses in both endemic and epidemic areas [2–4]. Trypanosomes are transmitted to their vertebrate hosts via saliva from both male and female tsetse flies as they take blood meals. There are only a limited number of methods to control the disease in the vertebrate hosts. Moreover, these types of disease control strategy can become unsustainable due to the evolution of resistance to trypanocide drugs [5]. Vector control thus remains an essential part of integrated disease management [6]. The sterile insect technique (SIT) is one of the few tsetse control methods that are efficient at low population densities and so has been proposed as holding the most promise for non-chemical control measures [7]. However, the use of SIT against tsetse is not always ethically acceptable because unlike in mosquitos, males also feed and so could transmit the parasites. It is thus essential to improve our knowledge of the determinants of tsetse refractoriness to trypanosome infection to develop strategies that could be combined with SIT to result in effective vector control. One possibility that has been suggested is exploiting natural endosymbionts predicted to reduce the establishment of trypanosomes in tsetse [8].

Many endosymbionts have been reported [9, 10] in various tissues of tsetse flies but *Wigglesworthia glossinidia, Sodalis glossinidius* and *Wolbachia spp.* are the three major bacterial species that they harbour [11]. *Sodalis glossinidius* is found in the midgut [12–14], haemolymph, muscles, fat bodies, salivary glands [14], milk glands and reproductive system [11–15] and so could interact with multiple species of trypanosomes that are harboured in different tissues. Indeed, *Trypanosoma congolense* is distributed in various stages of its life cycle in tsetse in the proboscis, foregut, midgut and proventriculus while the life cycle of *T. brucei* occurs in the foregut, salivary glands and midgut [16]. The life cycle and development of *T. vivax* within tsetse is thought to occurs only in the proboscis parts [17] but could also occur in parts of the cibarium/oesophageal region [18]. *Sodalis glossinidius* can be transmitted between tsetse flies through transovarial transmission via haemolymph [14], vertical transmission to intrauterine larvae via milk gland secretions, and horizontal transmission during mating [19]. The functional role of *S. glossinidius* in tsetse flies has not been clearly defined [20]. However, the presence of *S. glossinidius* has been shown to be positively associated with that of trypanosomes in tsetse flies, possibly through lectin-inhibitory activity [21]. Chitinase from *S. glossinidius* breaks down chitin and produces N-acetyl-D-glucosamine [22], which inhibits lectin function in the flies. Feeding flies lectin inhibitory sugars has been demonstrated to allow trypanosomes to more easily penetrate into the midgut [21], suggesting a mechanism by which the presence of the endosymbionts could increase trypanosome establishment. There is also some experimental evidence: for example, *G. morsitans morsitans* that were treated with the antibiotic streptozotocin to eradicate *S. glossinidius* showed a decreased reproductive capacity, decreased longevity and 40% increased trypanosome refractoriness compared to untreated flies [23, 24]. Other experimental studies revealed that the correlation between *S. glossinidius* and the ability of trypanosome to infect tsetse flies is trypanosome species-specific. For example, Wamwiri et al. [25] demonstrated that teneral male *G. pallidipes* were about 6 times more likely to become infected by *T. b. rhodesiense* if they were infected with *S. glossinidius* while infection by *T. b. brucei* was not correlated with the presence of the endosymbiont. Wamwiri et al. [25] also showed that *T. congolense* infection was 1.3 times more likely in flies not showing the presence of *S. glossinidius*. Geiger et al. [26] demonstrated an association between the presence of specific *S. glossinidius* genotypes and tsetse midgut experimental infection success by *T. b. brucei* and *T. b. gambiense*. In contrast, in other studies, *S. glossinidius* has been reported to stimulate the immune function of tsetse flies and so to decrease the levels of trypanosome infection [23, 27–30]. The relative importance of the host immune response and the ability of trypanosomes to colonise also might vary by species of trypanosomes and/or species of flies but this has not been systematically tested.

In field surveys, results also have been mixed, with some showing an association between *S. glossinidius* and any trypanosomes present [31, 32], some showing associations only with some of the tsetse species studied [33, 34], and others finding no association in any of the species tested [35]. However, the studies used a range of methods for detection of trypanosomes in flies, which could affect the power to detect associations. Moreover, they tended to pool data across species to increase sample sizes. Using electron microscopy to detect *S. glossinidius,* Maudlin & Ellis [32] found an association between *S. glossinidius* and trypanosomes (data for *T. brucei* s.l. and *T. congolense* s.l. pooled together) in both *G. pallidipes* and *G. morsitans* from a single site in Zimbabwe. Using a DNA probe to detect *S. glossinidius,* Maudlin et al. [33] found an association between *S. glossinidius* and trypanosomes (data for *T. vivax* and *T. congolense* s.l. pooled together) in *G. palpalis* but no association in *G. nigrofusca* or *G. pallicera* from Liberia. Other studies have used more sensitive PCR-based assays to detect *S. glossinidius* but still tended to pool data across species of trypanosomes [31, 34, 35]. Farikou et al. [31] reported a significant association between the presence of *S. glossinidius* and most trypanosome species identified in *G. palpalis palpalis* from two sites in Cameroon. In a study focused on tsetse populations in Kenyan coastal forests, Wamwiri et al. [34] found an association between *S. glossinidius* and trypanosomes (data pooled for *T. brucei. Brucei*, *T. vivax*, *T. congolense* and *T. simiae*) in *G. pallidipes* from a single geographic area but not in *G. austeni*. No association was reported in *G. brevipalpis*, *G. pallidipes* and *G. morsitans* from Zambia (trypanosome data pooled for *T. brucei* s.l., *T. b. rhodesiense*, *T. godfreyi, T. congolense* s.l. and *T. simiae*) [35].

In some of the studies mentioned above [33, 34], data collected from separated (dozens to hundreds of km) sites were also pooled for analysis. As such, geographical heterogeneity could be a confounding variable in the *S. glossinidius*-trypanosome association tests performed. Other variables such as sex and age of the flies, which could influence both *S. glossinidius* and trypanosome prevalence in tsetse populations [19, 25, 36–38], also were not explicitly considered in these studies. A detailed study is lacking that combines traits of the flies with the prevalence of both the endosymbiont and various species of trypanosomes across different geographic regions. Because *S. glossinidius* can be transmitted to tsetse both vertically and horizontally [19], sex and age of the tsetse flies could affect prevalence. For example, a decrease in *S. glossinidius* prevalence was found with increasing age of *G. pallidipes* in a time series experiment: the percentage of infection on days 7, 14, 21 and 28 post inoculation were 80.0, 86.4, 65.4 and 74.1, respectively [25]. However the role of sex varied by species of fly: males showed higher *S. glossinidius* prevalence than females, but no difference was found between male and female *G. m. morsitans* [35]. On the other hand, transmission of *S. glossinidius* from male to female flies during mating [19] could lead to higher levels of the bacteria in older females. Nevertheless, biological traits of tsetse flies have not been investigated systematically to tease out whether *S. glossinidius* presence itself enhances opportunities for trypanosome infection or whether the same factors that make prevalence of *S. glossinidius* more likely also make trypanosome infection more likely.

The overall aim of this study was to assess whether the presence of *S. glossinidius* predicts the relative prevalence of trypanosomes when taking into account other potentially confounding effects. Specifically, our objective was to test whether the presence of *T. vivax*, *T. congolense* and *T. brucei* in *G. austeni*, *G. brevipalpis*, *G. longipennis* and *G. pallidipes* from Kenya was associated with geographic location, species, sex or age of flies, the presence of the endosymbionts, or their interactions. We used both univariate (Generalised Linear Models, GLMs) and multivariate (Multiple Correspondence Analysis) methods to interpret patterns of association. We hypothesise that the discrepancy between previous studies on whether *S. glossinidius* presence is associated with trypanosome prevalence or not is due to not considering biological drivers that could affect both the symbiont and parasite (e.g. geographic location, host community composition, vector and parasite species composition), which could result in false predictions about causative associations.

## Results

### Distribution of trypanosomes and *S. glossinidius* in tsetse flies

**Fig. 1:**
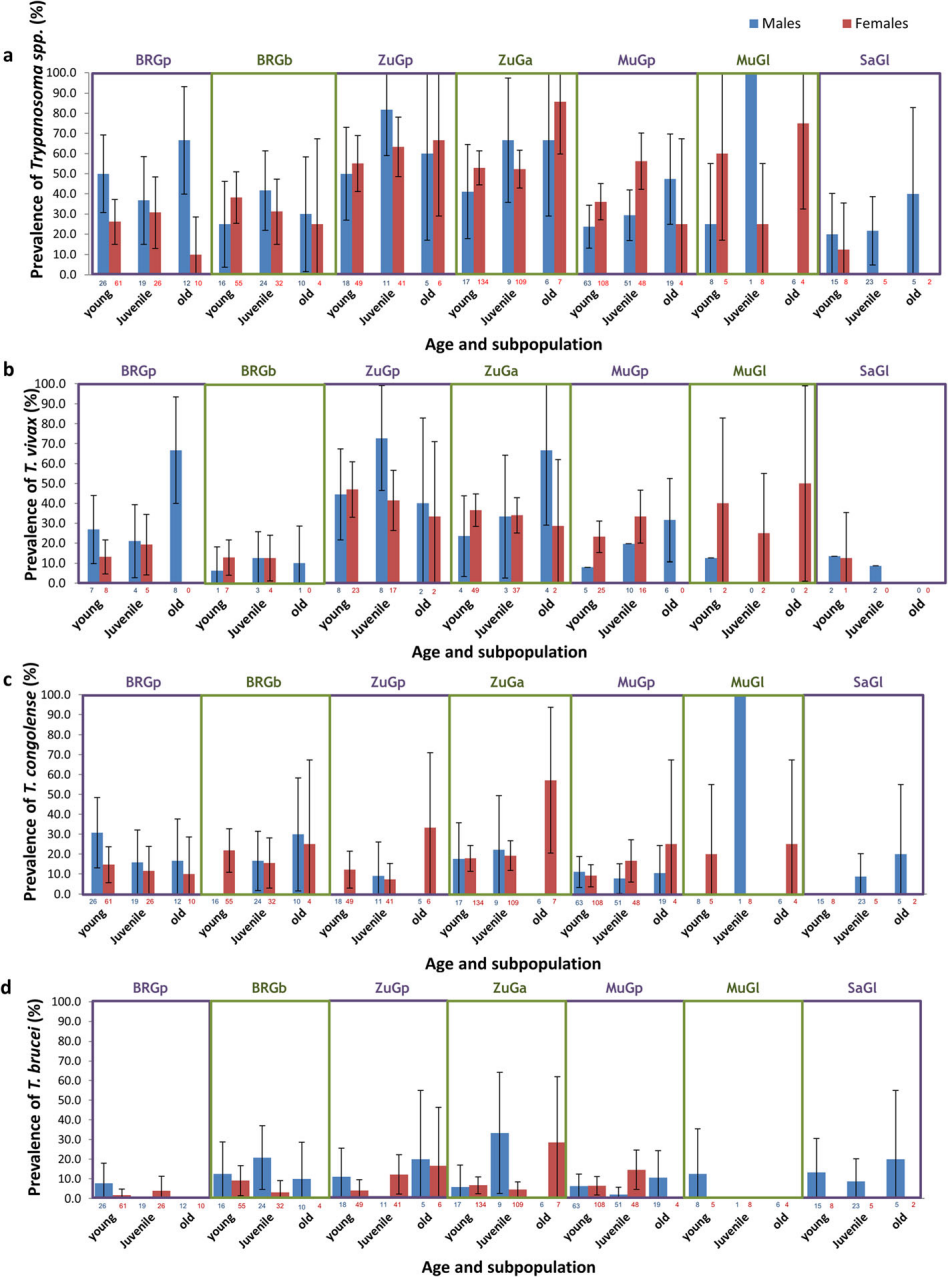
Prevalence of trypanosomes in male (blue) and female (red) tsetse flies with different ages in seven subpopulations. Prevalence in young, juvenile and old flies for (**a**) *Trypanosoma spp*., (**b**) *T. vivax*, (**c**) *T. congolense* and (**d**) *T. brucei*. BRGp: *G. pallidipes* from Buffalo Ridge; BRGb: *G. brevipalpis* from Buffalo Ridge; ZuGp: *G. pallidipes* from Zungu Luka; ZuGa*: G. austeni* from Zungu Luka; MuGp: *G. pallidipes* from Mukinyo; MuGl: *G. longipennis* from Mukinyo; SaGl: *G. longipennis* from Sampu. 95% CI error bars are represented. Total numbers of tsetse flies in each category are shown at the base of each bar

Overall, out of 1090 ‘head plus proboscis’ (HP) samples, 42.1% tested positive for trypanosomes based on the general ITS1 primers; 33.2% showed amplification of only a single positive band and 8.9% showed evidence for multiple infections (with up to four different species of trypanosomes present in single flies). The most common species were *T. vivax*, *T. congolense* and *T. brucei* but *T. simiae* and *T. godfreyi* were found at low frequency in some populations. Two subspecies of *T. congolense* (*T. c*. savannah and *T. c.* kilifi) were found, but they were combined for the statistical analyses, to increase power. Prevalence of the various trypanosome species varied by site of sampling, age and sex of tsetse flies (Fig. 1; Additional file 1).

Sequencing and BLAST analysis confirmed that the Hem primers amplified the entire coding sequence of the Hemolysin gene from *S. glossinidius*, with a single bp polymorphism among the 12 sequences obtained and one haplotype showing 100% match to the full genome sequence from *S. glossinidius str. Morsitans* (AP008232.1). For GPO1, three haplotypes were identified among the 20 sequences obtained but all showed at least 98% similarity to the pSG2 plasmid from *S. glossinidius (*AP008234.1).

**Fig. 2:**
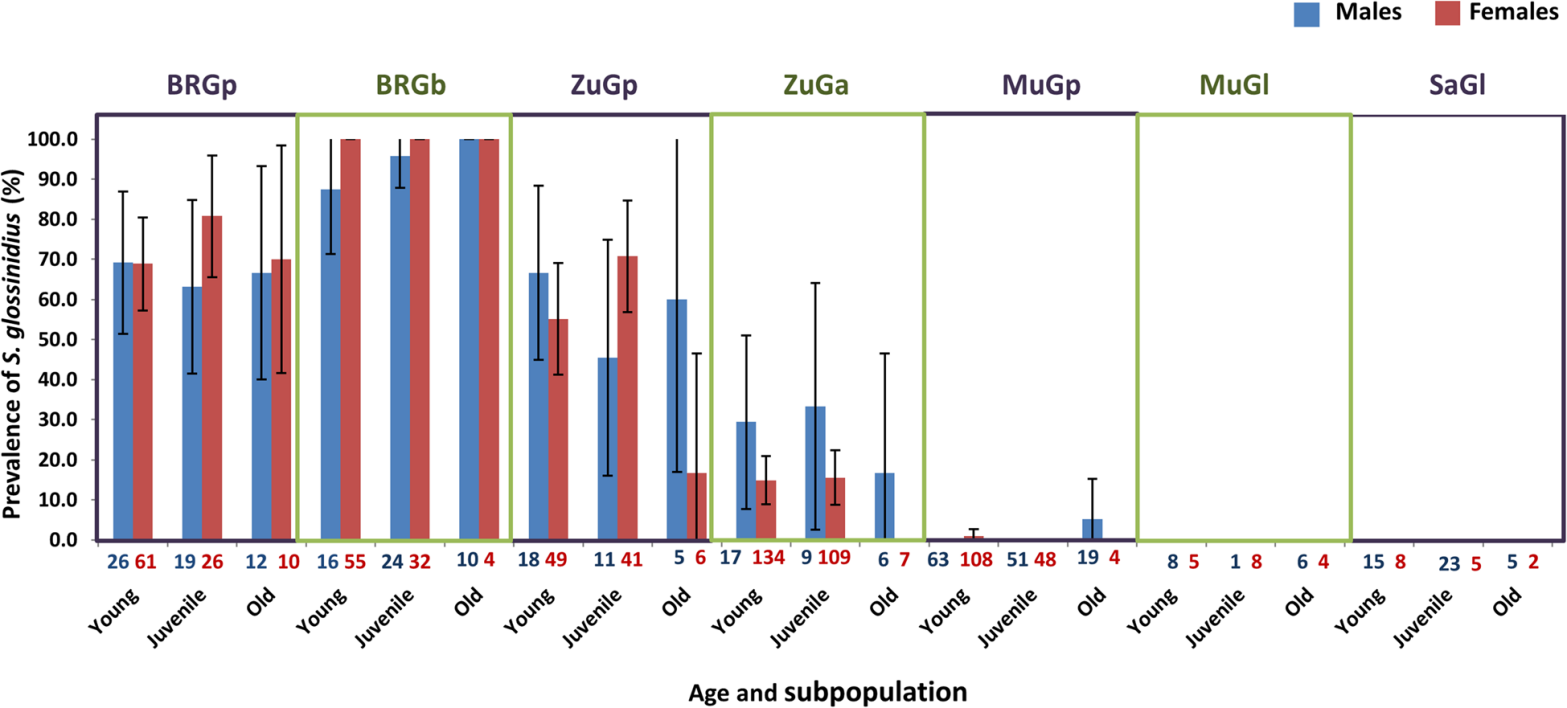
Prevalence of *S. glossinidius* in male (blue) and female (red) tsetse flies with different ages in seven subpopulations. Prevalence in young, juvenile and old flies. BRGp: *G. pallidipes* from Buffalo Ridge; BRGb: *G. brevipalpis* from Buffalo Ridge; ZuGp: *G. pallidipes* from Zungu Luka; ZuGa*: G. austeni* from Zungu Luka; MuGp: *G. pallidipes* from Mukinyo; MuGl: *G. longipennis* from Mukinyo; SaGl: *G. longipennis* from Sampu. 95% CI error bars are represented. Total numbers of tsetse flies in each category are shown at the base of each bar

Based on screening with the Hem primers, the overall prevalence of *S. glossinidius* from all 1090 tsetse samples was 34.0% but prevalence varied in relation to tsetse sex, age, species and sampling site (Fig. 2). Nearly all *G. brevipalpis* showed the presence of *S. glossinidius* whereas the presence of the endosymbiont in *G. pallidipes* varied between site, age class and sex, with an apparent interaction between site and the other factors for samples from the Shimba Hills National Reserve (SHNR) (Buffalo Ridge and Zunga Luka; Fig. 2). Prevalence was much lower in *G. austeni* sampled from this region than for the other two species. There was very low prevalence of *S. glossinidius* in the *G. pallidipes* sampled from the Nguruman region (only two positive flies) and none of the *G. longipennis* samples tested positive for *S. glossinidius* (Fig. 2).

**Fig. 3:**
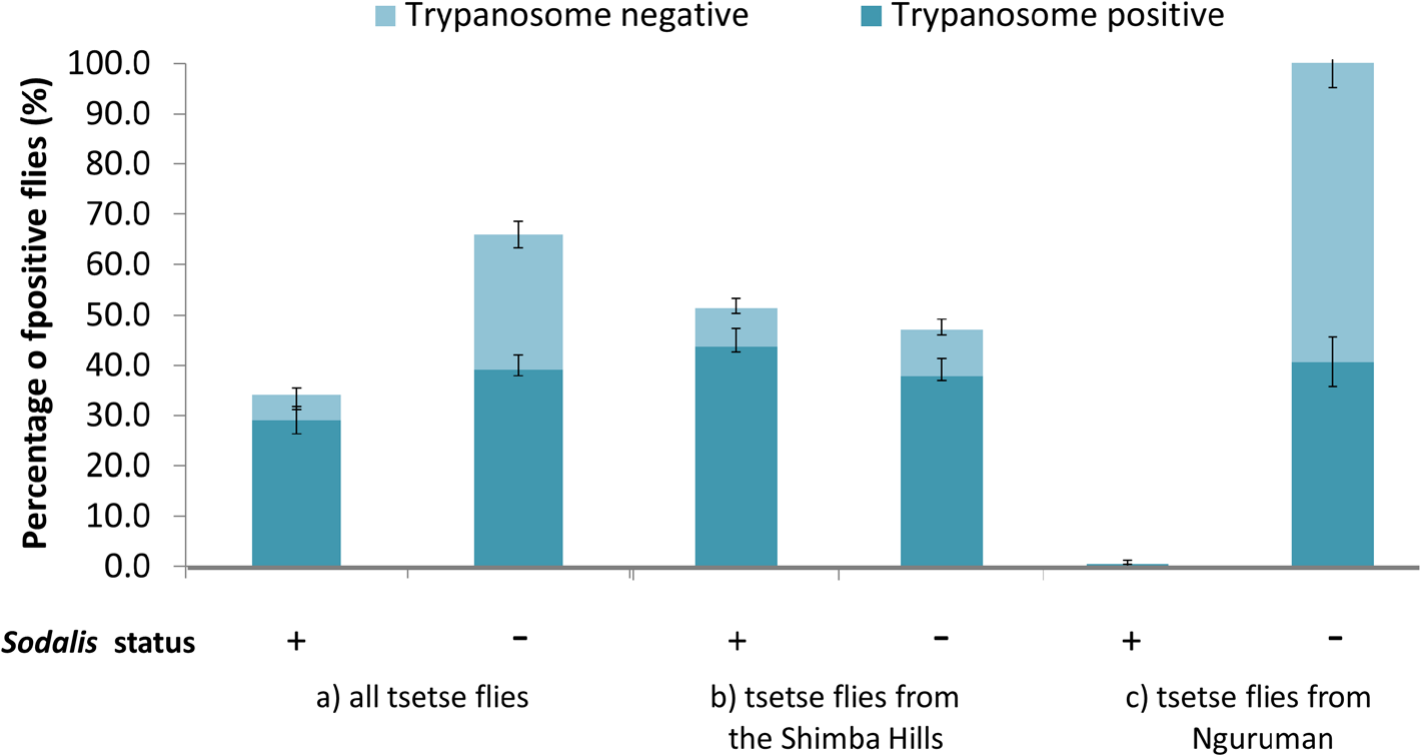
Comparison of *Trypanosoma* spp. and *S. glossinidius* screening results in tsetse flies. Histograms indicate the percentage of *S. glossinidius*-positive flies for: a) all tsetse flies (*N* = 1090 samples); b) the Shimba Hills National Reserve (*N* = 707 samples); and c) Nguruman (*N* = 383 samples)

Considering the raw association between *S. glossinidius* presence and trypanosome presence, 29% of the samples screened were positive for both but 39% were positive for trypanosomes and negative for *S. glossinidius* (Fig. 3). For samples from the SHNR, there was not much difference in the proportion of flies that tested positive for trypanosomes and that showed presence (44%) or absence (39%) of the endosymbiont. Although only two samples from Nguruman tested positive for *S. glossinidius,* 41% of samples tested positive for trypanosomes.

### Association between trypanosome prevalence, *S. glossinidius* presence and tsetse characteristics

#### Generalised linear models

Using likelihood ratio tests for model selection in GLM analyses, the final model for the presence of any species of trypanosome included age (χ^2^ = 4.65, *P*-value = 0.0310) and a two-way interaction between subpopulation and sex (χ^2^ = 18.94, *P*-value = 0.0043) (GLM Model 1; Table 1), with no effect of *S. glossinidius* status. It was clear that probabilities of trypanosomes presence in tsetse flies in all subpopulations increased when age increased, and some but not all subpopulations showed higher numbers of trypanosome present in females than in males (Additional file 2).

**Table 1:** Summary of the best-fitting GLM models when presence or absence of trypanosomes was considered as the response variable

Model Number	Explanatory variables of best fitting models	Full models		Best Fitting models	
		df	AIC	df	AIC
Model 1 (*Trypanosoma* spp*.*)	age + subpopulation * sex	44	1464	15	1432.5
Model 2 *(T. vivax)*	subpopulation + sex * age	44	1212.2	16	1176.3
Model 3 *(T. congolense)*	sex * *S. glossinidius* status * age + subpopulation	44	913.4	8	885.35
Model 4 (*T. brucei)*	subpopulation * sex * age + subpopulation * sex * *S. glossinidius* status + subpopulation * age * *S. glossinidius* status and sex * age * *S. glossinidius* status	44	588.8	42	584.8

A * *B* interaction between A and B, *df* degrees of freedom, *AIC* Akaike Information Criterion, Model 1 showed that only an interaction between subpopulation and sex was significantly associated with the presence of trypanosomes in tsetse flies using post hoc tests, but the associations were different for each species of trypanosomes. Interactions involving subpopulation and various tsetse factors significantly influenced the presence of *T. vivax.* Interactions between sex and *S. glossinidius* status were significantly associated with *T. congolense* status. However, post hoc tests did not resolve which factors were significantly associated with *T. brucei* status

*T. vivax* presence was significantly associated with interactions between subpopulation and sex (χ^2^ = 18.90, *P*-value = 0.0043) and between sex and age (χ^2^ = 7.52, *P*-value = 0.0059), without involving *S. glossinidius* presence (Model 2; Table 1). A three-way interaction among sex, age and *S. glossinidius* status was significantly associated with *T. congolense* prevalence (Model 3; Table 1; χ^2^ = 6.84, *P*-value = 0.0089). The probability of *T. congolense* presence was predicted to increase for *S. glossinidius*-positive males with increasing age, but to decrease for *S. glossinidius*-positive females (Additional file 2). On the other hand, the absence of *S. glossinidius* in females tended to increase probabilities of *T. congolense* presence with age but decreased for *S. glossinidius*-negative males. However for *T. brucei* (Model 4; Table 1), the results were even more complicated, with three different three-way interactions involving status: a three-way interaction among sex, age and *S. glossinidius* status (χ^2^ = 7.94, *P*-value = 0.0048); a three-way interaction among subpopulation, age and *S. glossinidius* status (χ^2^ = 13.84, P-value = 0.0031); and a three-way interaction among subpopulation, sex and *S. glossinidius* status (χ^2^ = 9.27, P-value = 0.0097). Although it is not possible to interpret the biology of such complex interactions, predicted values demonstrate the overall directions of associations (Additional file 3). For example, both species of tsetse flies (*G. pallidipes* and *G. brevipalpis*) from Buffalo Ridge tended to show decreased probabilities of *T. brucei* presence with age but this varied by sex and *S. glossinidius* status: males showed a higher probability of *T. brucei* presence than females and lower values in older flies, but an association with age was only found for females that lacked *S. glossinidius*. In contrast, even though *G. pallidipes* was also found at Zungu Luka, different patterns of relationships were found among sex, age and presence of *S. glossinidius* in relation to probabilities of the presence of *T. brucei*.

#### Multiple correspondence analysis

**Fig. 4:**
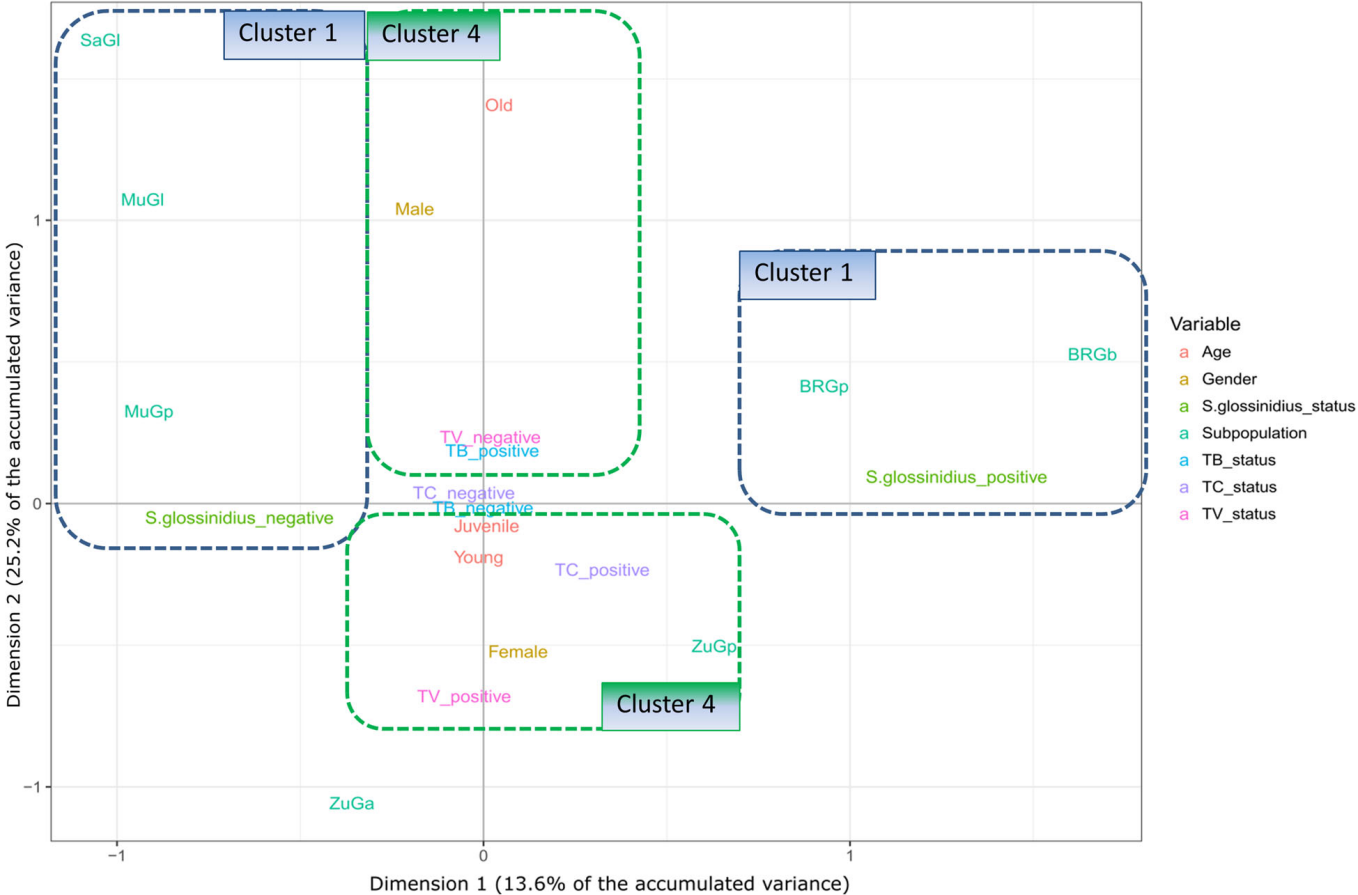
Dimensions 1 and 2 of the Multiple Correspondence Analysis 2 showing the relationships among infections of each of the three main species of trypanosomes in relation to *S. glossinidius* status and biological traits of tsetse flies. Sex (male and female), age (young, juvenile and old) and *S. glossinidius* status of the seven subpopulations were plotted for describing associations with the trypanosome status (for each of the three main species of trypanosomes) of tsetse flies. TV: *T. vivax*; TB: *T. brucei*; TC: *T. congolense*. BRGp: *G. pallidipes* from Buffalo Ridge; BRGb: *G. brevipalpis* from Buffalo Ridge; ZuGp: *G. pallidipes* from Zungu Luka; ZuGa: *G. austeni* from Zungu Luka; MuGp: *G. pallidipes* from Mukinyo; MuGl: *G. longipennis* from Mukinyo; SaGl: *G. longipennis* from Sampu

Given the complexity of interactions found in the Generalised Linear Models (GLM) for the presence of any species of trypanosome, two-dimensional Multiple Correspondence Analysis (MCA) was used to more clearly demonstrate relationships among levels of each factor considered (Additional files 4 and 5). For the first two principal components, using either overall trypanosome species presence (Additional files 5 and 6) or including each trypanosome species separately (Fig. 4; Table 2), clearly demonstrated that the presence of *S. glossinidius* (resolved primarily along the PC1 axis; with 86% of the variation explained in this dimension) was explained by a different combination of factors than presence of trypanosome (for which PC1 explained very little of the variation). In particular, subpopulation appeared to the primary driver of *S. glossinidius* presence whereas trypanosome presence was explained more by the biology of the flies (sex and age, which were most strongly resolved along PC2). The third principal component did not explain any of the variation in *S. glossinidius* presence but explained more of the variation in trypanosome status than the other two dimensions (Table 2; Additional file 6). Variation among subpopulations was partitioned among all three dimensions but most strongly in PC1 (87% of the variation).

**Table 2:** Adjusted *Eta*^2^ for the combination of variables in dimensions 1–3 in Multiple Correspondence Analysis 2

Variables	Dimension 1	Dimension 2	Dimension 3
*S. glossinidius*_status	0.860	0.005	0.005
subpopulation	0.867	0.585	0.423
sex	0.018	0.543	0.018
age	0.000	0.203	0.058
*T. congolense*	0.017	0.009	0.255
*T. brucei*	0.000	0.003	0.154
*T. vivax*	0.001	0.160	0.307

## Discussion

### Association between trypanosome prevalence and *S. glossinidius* presence in tsetse flies

Our results clearly demonstrate that prevalence of trypanosomes in tsetse vectors is influenced by multiple factors, and suggest that the previously mixed results found for an association with the endosymbiont *S. glossinidius* [31–35] could thus be confounded by differences in the age distribution, sex ratio, or species of both vectors and parasites at different sites. In fact, if we had ignored differences among geographic regions or species of flies, we might have concluded that there was an over-representation of flies that were both trypanosome and *S. glossinidius*-positive (Figs. 1 and 2). This was not the case if only Buffalo Ridge region was considered, which showed a high prevalence of both parasites and endosymbiont but showed no association between the two. This observation is consistent with the negative correlation, described by Baker et al. [39], between *S. glossinidius* prevalence and the strength of the *S. glossinidius*/trypanosome association. Similarly, *S. glossinidius* was rare in the Nguruman region, but there was still a relatively high proportion of individuals that tested positive for trypanosomes. Moreover, across all regions, flies that lacked *S. glossinidius* showed a nearly equal chance of testing positive for trypanosomes as those where it was present. It was also not the case that sites with the highest trypanosome prevalence were also those with the highest *S. glossinidius* presence: Buffalo Ridge showed the highest prevalence of *S. glossinidius,* but Zungu Luka showed the highest prevalence of trypanosomes. Our results thus do not support the hypothesis that the presence of trypanosomes has a strong association with this particular endosymbiont. While experimental tests would be necessary to rule out the possibility that the endosymbiont enhances uptake of the parasites [22, 23], the presence of *S. glossinidius* does not appear to be a useful factor for predicting infection with trypanosomes, at least in the geographic region sampled.

Lack of association between the parasites and *S. glossinidius* was also evident in the GLM analyses. The presence of the endosymbiont was identified as a significant factor only in three-way interactions with other variables when *T. brucei* and *T. congolense* were analysed separately, whereas no significant associations involving *S. glossinidius* were found for *T. vivax* or when all species of trypanosome were combined. The relative importance of the endosymbiont thus could vary by species of trypanosomes, as suggested in previous studies, which reported significant associations of *S. glossinidius* with *T. b. rhodesiense* and *T. congolense* but not with *T. b. brucei* [25]. Regarding *T. vivax*, the complete lack of association with *S. glossinidius* in our dataset is possibly a consequence of the *T. vivax* life cycle. Indeed, *T. vivax* is classically thought to complete the tsetse part of its life cycle only in the fly mouthparts [40], where it is possibly never in interaction with *S. glossinidius*. Nevertheless, our results highlight the importance of considering other potentially confounding factors. For example, the role of *S. glossinidius* in affecting immune mechanisms might be different in males and females and in different species of flies from different geographic regions. A previous study found that *S. glossinidius* enhances trypanosome harbouring in males but induces trypanosome defence in females, although the mechanisms remain unclear [16]. *S. glossinidius* could thus have an influence on trypanosome prevalence, but its influence could differ by species or age of the flies, consistent with biological predictions based on the site of harbouring of trypanosomes [16].

It is intriguing that no *S. glossinidius-*positive samples were found in *G. longipennis*, which was only found in the Nguruman region, and that prevalence of the endosymbiont was also lower in the widespread *G. pallidipes* in this region compared to the SHNR. Using presence of *S. glossinidius* as a binary response variable and without considering trypanosome prevalence, we found that “subpopulation” was the only factor significantly associated with the endosymbiont (Additional Analyses A in Additional file 7). We also tested *G. pallidipes* on its own (Additional Analyses B in Additional file 7) so that we could disentangle the effects of tsetse species from site; site was again the only significant variable, which was driven by significantly (*p* < 0.001) more *S. glossinidius* found at each of the SHNR sites compared to Mukinyo (Nguruman). The presence of *S. glossinidius* might thus be explained more by habitat than by other factors. Nguruman is described as acacia woodland, scattered bushes and open grasslands [41], surrounded with open savannah [42], while the SHNR is tropical evergreen seasonal lowland rainforest [43]. The different forest types are characterised by different ranges of temperature, humidity and available hosts of tsetse flies. Such factors might influence the presence of the bacteria, and affect the frequency of horizontal transmission. It is also possible that there is genetic variation among strains of *S. glossinidius* and that our primers did not amplify the dominant genotypes found in Nguruman. However, in a pilot analysis, we had tested two other sets of primers, which showed similar results [44]. Similarly, previous studies have reported the absence of *S. glossinidius* in some *G. fuscipes fuscipes* populations in Kenya and Uganda [10, 45]. However, a recent study on *G. fuscipes fuscipes*, *G. m. morsitans* and *G. pallidipes* populations in Uganda, involving 16S rRNA deep sequencing and qPCR to detect *S. glossinidius*, showed that all the flies were infected but that *S. glossinidius* was present at a very low density within flies [46]. In the same study, *S. glossinidius* detection by conventional PCR was also performed. While 16S rRNA deep sequencing detected *S. glossinidius* in every fly analysed, the estimation of the *S. glossinidius* prevalence using conventional PCR was < 54%. *Sodalis glossinidius* prevalence estimated by PCR is thus likely associated with *S. glossinidius* density in each fly. As a consequence, the very low prevalence of *S. glossinidius* reported in the present study in *G. pallidipes* from Nguruman and in *G. longipennis* could be a reflection of low density rather than the absence of *S. glossinidius* in some populations or tsetse species.

### Factors affecting the presence of trypanosomes in tsetse flies

The GLM analyses supported the conclusion that subpopulation (classified by species of tsetse flies and sampling site), age and sex of flies were all associated with the presence of trypanosomes in the tsetse flies but that the nature of the associations varied by species of trypanosome (Table 1). *Trypanosoma brucei* showed the most complex interactions, but this was also the parasite with the lowest prevalence so there might not have been sufficient power to resolve the main factors driving variation.

Overall, our results emphasise the importance of not considering factors in isolation. In general, across all subpopulations, the apparent effects of site and tsetse species were confounded by differences in sex and age distributions and potential sampling biases related to the species of tsetse found at each site. For example, at one site (Zungu Luka) where more than one species of tsetse was present, the prevalence of trypanosomes was higher in *G. pallidipes* than *G. austeni*. Since the lifespan of *G. pallidipes* females has been found to be longer than that of *G. austeni* [34] and a long lifespan of tsetse flies could increase the risk of trypanosome exposure [37, 38] and infection, this could contribute to the observed differences. Moreover, *G. pallidipes* has a broader distribution than *G. austeni* and so might be more likely to encounter a wider range of host species, including domesticated animals. Nevertheless, across all populations, the highest trypanosome prevalence was actually found in *G. austeni*, followed by *G. pallidipes*, *G. brevipalpis* and *G. longipennis* but this could have been influenced by sex since there was a stronger bias towards females in the ZuGa subpopulation compared to the others. Since *G. austeni* was only found at Zungu Luka, it is not possible to separate which factor (site, species of tsetse or their age or sex) is most important for trypanosome susceptibility but it emphasises how misleading interpretation of results could be if considered in isolation. To test the effects of site separately from tsetse species, we also considered associations with the presence of any trypanosome species separately for *G. pallidipes*, which was sampled from three sites (BR, Zu and Mu). We found a significant interaction between site and fly sex but no effects of age or *S. glossinidius* (Additional Analyses C in Additional file 7).

Relative prevalence of trypanosomes across sites and fly species also differed by species of trypanosomes. Although *G. austeni* on average showed a higher prevalence of trypanosomes than *G. pallidipes*, this was driven by low prevalence in the latter at two sites (Buffalo Ridge and Mukinyo): at Zungu Luka *G. pallidipes* actually showed higher prevalence than *G. austeni*, particularly for *T. vivax.* Similarly, *G. brevipalpis* was the only species for which a higher infection was found for *T. congolense* than *T. vivax*, but it was found at only a single site (Buffalo Ridge), where relatively high infection with the former was also found in the other species present (*G. pallidipes*). These results are consistent with a previous report that tsetse flies in the *Morsitans* group (*G. austeni* and *G. pallidipes*) are better hosts for trypanosomes than the *Fusca* group (*G. brevipalpis* and *G. longipennis*) [47]. Another study reported that *G. austeni* were more susceptible to trypanosome maturation than *G. brevipalpis* [48]. However, more systematic sampling would be required to test these associations rigorously. Nevertheless, our results strongly suggest that geographic location, tsetse species, age and sex could be stronger drivers of trypanosome prevalence than the presence of *S. glossinidius*.

### Multivariate visualisation of associations

Since it is not possible to biologically interpret such complex interactions in GLM models, multivariate analyses, such as the MCA used here, provide an appropriate means of visualising correlations among factors. Graphical MCA supported conclusions from the GLM analyses that presence of *S. glossinidius* was distinctly correlated to only particular subpopulations of tsetse flies while trypanosome infection was correlated more with sex, age and subpopulation than *S. glossinidius* status. The association of *T. brucei* with sex and age was different than for *T. vivax* and *T. congolense*. *T. brucei*-positive flies tended to be found in old and male flies whereas the other two were more common in young female flies. The environment of salivary glands belonging to female *G. m. morsitans* and *G. pallidipes* is more inhospitable for *T. brucei* than males and could be a result of higher effective immune response in the former [36]. Although these patterns were quite weak, and no strong predictions were suggested about the association of each species of trypanosomes with tsetse factors (including *S. glossinidius* status), we suggest that multivariate analyses such as this could have more potential for resolving complex associations and for designing more targeted studies to identify risk factors at a more local scale.

### Potential limitations associated with PCR-based *Trypanosoma* spp. identification

While our results clearly question whether *S. glossinidius* would make a good target for altering refractoriness of flies to trypanosomes, two issues associated with the utilisation of a PCR-based detection and identification approach as utilised in this study should be addressed. First, PCR detects trypanosome DNA introduced in the tsetse bloodmeal, which can remain stable in tsetse tissues for some time after the death of trypanosomes. This could lead to false positive conclusions about ‘infections’ and thus prevalence estimates that would be higher than the true prevalence. We have decreased the problem associated with this issue by investigating HP samples, which contain the foreguts. On ingestion of a contaminated blood meal, trypanosomes travel through the foregut quickly into the midgut (Chinery, 1965). The traditional other option to PCR-based detection of trypanosome is microscopy. However, microscopy is less sensitive than PCR and is associated with high levels of false negatives and thus prevalence estimates that are lower than the true prevalence [49]. Moreover, trypanosome identification by microscopy is not very accurate and cannot differentiate between closely related species [50]. The second issue is that the detection of trypanosomes in HP samples does not demonstrate whether their full life cycle has occurred and that they are able to be transmitted. Our data thus correspond to presence/absence of trypanosome in HP samples and not exactly to infection of tsetse by trypanosomes. However, experimental infections have revealed that trypanosome DNA cannot be amplified by the ITS1 PCR used in this study more than 24 h post infected blood meal in HP samples (pp. 122–123 and Appendix A.8 of [44]). Considering that wild tsetse feed ≥3 days (p. 328 [47]) we believe that the presence/absence of trypanosome in HP samples measured in this study is a good proxy to trypanosome infection.

## Conclusions

Although we did not find a strong association between trypanosome status and *S. glossinidius* status, *T. congolense* and *T. brucei* were significantly associated with the presence of *S. glossinidius* in some analyses, but only in complex interactions with other tsetse-specific factors. It is thus important not to study potential associations in isolation; these results emphasise that the factors determining whether tsetse flies are infected with trypanosomes are complex and considering only simple associations might not be informative enough to guide management programs. A better understanding of host-feeding patterns of tsetse flies would also be important to assess in relation to risk of trypanosome transmission between hosts.

## Methods

### Sample collection and DNA purification

Tsetse flies were randomly sampled in 2012, between 15 June and 15 July from Buffalo Ridge (BR, − 4.242^°^, 39.436^°^) and Zungu Luka (Zu, − 4.339^°^, 39.264^°^, 22 km from BR) in the Shimba Hills National Reserve (SHNR), and between 10 and 14 August from Mukinyo (Mu, − 1.836^°^, 36.086^°^) and Sampu (Sa, − 1.890^°^, 36.075^°^, 6 km from Mu) in Nguruman. Flies were trapped using improved NG2G traps [51] baited with acetone and > 3 weeks old cow urine. The collection cage usually made of netting at the top of the trap was replaced by a 1 L plastic water bottle attached to the trap with a ~ 45^°^ angle and containing 95% ethanol (Additional file 8). Because they were collected in ethanol, tsetse flies could be recovered from the traps in very good condition for subsequent analysis. In the modified cages, the trapped tsetse flies were in contact with the ethanol a few seconds after entering the cage. This limits to a minimum the damage caused to wings when flies move around alive in conventional netting cages. The modified cages are thus particularly useful to collect tsetse flies that can afterwards be aged using the wing fray technique [52]. The traps were emptied every day around 10.30 am and/or 5.00 pm [53]. Whole flies were preserved in 95% ethanol and stored at 4 °C.

The tsetse flies collected were identified based on their specific morphology [54] and based on previous reports describing their presence or absence in Nguruman (*G. pallidipes* and *G. longipennis*) and the SHNR (*G. pallidipes*, *G. brevipalpis* and *G. austeni*) [55]. *Glossina pallidipes* was found at all sites, but only three subpopulations were defined because Mukinyo and Sampu (which are located 6 km apart) correspond to a single population [56, 57]: Buffalo Ridge (BRGp); Zungu Luka (ZuGp) and Mukinyo (MuGp). *G. longipennis* was found only in the Nguruman region (Mukinyo and Sampu) and so, two subpopulations were defined: MuGl and SaGl. The other two species were only found at a single site: *G. austeni* at Zungu Luku (ZuGa) and *G. brevipalpis* at Buffalo Ridge (BRGb). All individuals trapped were used for the downstream analyses for three of the species (282 *G. austeni*, 141 *G. brevipalpis* and 90 *G. longipennis*) but only a subset of the *G. pallidipes* collected was used (293 from Nguruman and 284 from the SHNR) since they were found in high numbers at all sites. A total of 1090 tsetse flies were thus analysed. The sex of all flies was physically determined by examination of the external sex organs on the ventral part of the abdomen under a stereoscope. The relative age of tsetse flies was determined based on the wing fray score described by Jackson [52]. The principle is that wing damage increases with age; flies were grouped into “young” (wing fray score 1–2.5); “juvenile” (3.0-4.0) and “old” (4.5–6.0) age categories based on the average score between the two wings for each individual (see details on pp. 74–76 of Wongserepipatana [44]).

To determine the most appropriate tissues to amplify both trypanosomes and *Sodalis*, ‘head plus proboscis’ (HP) and ‘abdomen’ (AB) were dissected out from all flies for trypanosome (*T. vivax, T. brucei* and *T. congolense*) and *S. glossinidius* screening [44]. Scalpel blades and Petri dishes used for dissections were changed for each individual to minimise cross-contamination among samples. Forceps were also cleaned with 10% bleach (soaked for > 3 min), water and double distilled water. Each sample was frozen using liquid nitrogen and crushed with a single-use micropestle (Starlab.Co.UK); used micropestles were soaked in 10% bleached overnight and thoroughly rinsed in water and double distilled water before being reused. Only the external part of the tube containing the sample was in contact with liquid nitrogen, not the sample itself. DNA extractions were conducted separately for HP and AB parts using DNeasy^®^ blood and tissue kits (Qiagen Inc., Paisley, UK), following the manufacturer’s instruction for animal tissues [58], except the final elution volume was altered to 60 and 100 μl buffer for HP and AB, respectively. A negative extraction control was included for each set of 50 samples, to check for contamination in reagents. A pilot study, not presented here (but see details on pp. 87–88 of Wongserepipatana [44]), revealed that the HP DNA samples were more suitable than AB DNA samples. Indeed, compared to AB DNA samples the HP DNA samples allowed the detection of *S. glossinidius* as well as of all tsetse-transmitted trypanosome species, irrespective of their life cycle in tsetse. Moreover, experimental infections have revealed that trypanosome DNA cannot be detected by the ITS1 PCR more than 24 h post infected blood meal in HP samples while detection is still possible in AB samples up to 8 days post infected blood meal (pp. 122–123 and Appendix A.8 of Wongserepipatana [44]).

### Trypanosome and *S. glossinidius* identification using PCR

Trypanosome detection and identification were performed using the PCR assay developed by Njiru et al. [59] that amplifies the internal transcribed spacer 1 (ITS1) region of the ribosomal DNA of all known trypanosome species (Additional file 1). The PCR products of each species have been described as having their own specific sizes, as follows: 697 bp for *T. congolense* savannah (Tcs); 600 bp for *T. congolense* kilifi (Tck); 476 bp for *T. brucei* (Tb); 397 bp for *T. simiae* (Ts); 273 for *T. godfreyi* (Tg); and 250 bp for *T. vivax* (Tv) [59, 60]. Thus, this PCR assay has conventionally been used for trypanosome species identification. PCR was carried out in 10 μl reaction mixtures containing 1 μl of 10X Custom PCR Master Mix (Thermo Scientific, ABgene^®^ UK. The final 1X concentration for that Custom PCR Master Mix corresponds to 45 mM Tris-HCl (pH 8.8), 11 mM (NH_4_)_2_SO_4_, 4.5 mM MgCl_2_, 0.113 mg/ml BSA, 4.4 μM EDTA, 1 mM each of dATP, dCTP, dGTP and dTTP, 10 μM ITS1 primers [59], 1 μl DNA template (20–200 ng) and 1 unit of *Taq* DNA polymerase (Thermo Scientific). PCR conditions for ITS1 amplification were: 94 °C for 5 min; 35 cycles of 94 °C for 40 s, 58 °C for 40 s, 72 °C 90 s; and 72 °C for 5 min [59]. All sets of reactions included a negative control (distilled water) to check for contamination; the negative extraction control was also amplified for each set of extractions. In addition, DNA purified from cryopreserved mouse blood infected with trypanosomes (*T. b. brucei* strain STIB 247, *T. vivax* ILRAD V-34 and *T. congolense* savannah IL3000) was used as PCR positive control [58]. Reaction sets where the positive control did not amplify or where an amplification product was apparent in the negative control were repeated. Any DNA samples that were negative for trypanosomes were screened for *Glossina* DNA using *Glossina* ITS1 primers [44, 61] to check the quality of extracted DNA. Sequences were obtained from DNA samples that showed a single positive band for each species (*T. brucei, T. vivax, T. congolense* kilifi and *T. congolense* savannah) to confirm trypanosome species identification. Positive bands for each species (one each) were purified with QIAquick Gel Extraction Kits (Qiagen Inc., Paisley, UK) and cloned using TOPO^®^-TA Cloning Kits (Invitrogen Inc., Carlsbad, CA., USA). DNA from six plasmids of each clone was extracted with QIAprep Spin Miniprep Kits (Qiagen Inc., Paisley, UK). Plasmid DNA was then sequenced using M13 forward and M13 reverse primers by the University of Dundee DNA Sequencing Service (www.dnaseq.co.uk) using Applied Biosystem Big-Dye version 3.1 chemistry on an Applied Biosystem Model 3730 automated capillary DNA Sequencer. Chromatographs were manually corrected and compared using Sequencher, version 5.3 (Gene Codes Corporation, Ann Arbor, MI USA). The identity of the sequences was then determined by using the Basic Local Alignment Search Tool (BLASTn) to determine the closest match to available trypanosome species in GenBank. The percentage of identity was determined by the number of bases which matched exactly between the sequences from the samples and sequences available in Genbank but gaps were not counted and the measurement is relational to the shorter of the two sequences. This is because percentage overlap is not as meaningful for PCR fragments, where different authors may have used different primer sets for amplification.

The presence of *S. glossinidius* was determined by PCR with Hem primers (Additional file 9) [62], to target a gene encoding the haemolysin protein [63]. A detailed comparison of amplification products (see details in Chapter 3 of Wongserepipatana [44]) suggested that the use of this nuclear gene provided a more reliable assessment of prevalence than the other two commonly used primer sets (pSG2-Farikou [64], GPO1; [11]), which target different regions of the *S. glossinidius* pSG2 plasmid (Additional file 10) [44]. PCR reaction mixtures (10 μl) used 5 μl of Dream Taq Green PCR Master Mix (2X) (Thermo Scientific), 10 μM of forward and reverse primers and DNA template (20–200 ng). PCR cycles were: 94 °C for 2 min; 30 cycles of 94 °C for 30 s, 54 °C for 40 s and 72 °C for 60 s; and 72 °C for 7 min [62]. A *S. glossinidius*-positive control was included in all reaction sets, along with a negative PCR control. We did not have access to a *S. glossinidius* culture to prepare DNA that could be used as a positive control in the *S. glossinidius* detection PCR. To overcome this, we used tsetse from a laboratory colony known to be infected by *S. glossinidius*. Boucias et al. [65] had suggested that the *S. glossinidius* infection rate is high in the *G. pallidipes* IAEA colony originating from southeastern Uganda [66]. To confirm that the extracted DNA from the IAEA colony can be used as a positive control for the field samples, PCR products were amplified using Hem primers, and then the purified DNA fragments were sequenced to confirm the identity of amplification products. To further test whether the primers amplified *S. glossinidius* specifically, PCR products of 20 positive samples were cleaned using ExoSAP-IT PCR Clean-up Kits (GE Healthcare) and sequenced directly using the PCR primers. Sequences were obtained from Hem products from seven samples of *G. pallidipes* (Gp) and five samples of *G. austeni* (Ga) from Zungu Luka (Zu); and three samples of *G. brevipalpis* (Gb) and five samples of *G. pallidipes* from Buffalo Ridge (BR). For comparison, we also amplified and sequenced products from GPOI from the same individuals, using primers located in the noncoding region upstream of the repA1 protein and extended 767 bp into the start of the coding region. The results from both genes were analysed using BLASTn to confirm the identity of amplification products.

### Statistical analyses

Generalised linear models (GLMs), as implemented in the glm2 package (version 1.1.2) of the R statistical software programme (version 3.1.2) [67], were initially used to test for associations of amplification of any of the presence of *Trypanosoma* species with tsetse fly species, collection site, sex and age of tsetse flies, and the presence of *S. glossinidius* as explanatory variables. All pairwise and three-way interactions were also considered in the full initial model. Due to different species composition in each population and region, tsetse species and collection site were collapsed into a single variable, ‘subpopulation’. All tsetse variables were treated as fixed categorical effects: 1) subpopulation (seven levels); 2) sex (male and female); and 3) age (young, juvenile and old). The presence of trypanosomes and *S. glossinidius* were treated as binary variables (‘0’ or ‘1’ for presence or absence), so the binomial family was used to model the underlying error distribution of the response variable. In order to fit the best models, the variables from the full model (all explanatory variables and their interactions) were manually removed using a backwards elimination technique. Variables that did not significantly (alpha = 0.05) improve the fit of the model based on Likelihood Ratio Tests (LRTs) were excluded. Separate GLMs were also run using each of the three most common species of trypanosomes as response variables: *T. congolense*, *T. brucei* and *T. vivax*. In order to identify relationships within variables with multiple levels and for interactions that were found to be significant, the TukeyHSD Package *stats* version 3.4.0 command was applied to the best fitting model for post hoc comparisons. The predict() function in R was used to predict the probability of *S. glossinidius*-positive status for the best-fitting model for each response variable.

Given the potential complexity of interactions among the variables considered, Multiple Correspondence Analysis (MCA), as implemented in the FactoMineR package (version 1.30) and the ‘ggplot2()’ function was applied to graphically visualise associations among trypanosomes, tsetse biological traits and *S. glossinidius* in all collected flies. This type of multivariate analysis can provide clearer identification between multiple levels of compound categorical response variables than GLMs, where it is not possible to interpret higher than pairwise interactions. MCA 1 included five categorical variables, comprised of 1) presence or absence of *S. glossinidius*; 2) presence or absence of any *Trypanosoma spp.*; 3) subpopulation; 4) sex; and 5) age of tsetse flies. MCA 2 was the same but considered presence or absence of each of the main species of trypanosomes (*T. vivax*, *T. congolense* and *T. brucei*) separately. The parameter *Eta*^2^ is the correlation ratio and indicates the proportion of the total sum of squares that is explained by the predictor in each dimension. High *Eta*^2^ of each factor means strong correlations, which forms the basis of the resulting cluster analysis.

## Additional files

Additional file 1: **Table S1.** Presence/absence of trypanosomes and *S. glossinidius* in relation to tsetse location, species, sex and age. Since sites differed in the species composition of tsetse, “Subpop” refers to the combination of site and species for the generalised linear modelling. Presence or absence of individual trypanosome species was recorded but, due to the low prevalence of some, only *T. congolense* (combining the subspecies *T. c.* kilifi and *T. c.* savannah), *T. vivax* and *T. brucei* were included in separate statistical analyses. The variable “Tryps” included all species and subspecies present. (XLSX 66 kb)

Additional file 2: **Figure S1.** Probability of *T. congolense* presence in tsetse samples from the best-fitting model (Model 3). pos_Sodalis: *S. glossinidius*-positive status. neg_Sodalis: *S. glossinidius*-negative status. This analysis shows the significant three-way interaction between sex, age and *S. glossinidius* status. (PDF 93 kb)

Additional file 3: **Figure S2.** Probability of *T. brucei* presence in tsetse samples from the best-fitting model (Model 4), showing complex interactions. Predicted values are shown for males and females in the seven subpopulations: (a) *G. pallidipes* from Buffalo Ridge. (b) *G. brevipalpis* from Buffalo Ridge. (c) *G. pallidipes* from Zungu Luka. (d) *G. austeni* from Zungu Luka. (e) *G. pallidipes* from Mukinyo. (f) *G. longipennis* from Mukinyo. (g) *G. longipennis* from Sampu. (PDF 140 kb)

Additional file 4: **Figure S3.** Dimensions 1 and 2 of the Multiple Correspondence Analysis 1 showing the relationships between the trypanosome status of tsetse flies and their biological traits. Sex (male and female), age (young, juvenile and old) and *S. glossinidius* status (negative and positive) of the seven subpopulations were plotted for describing associations with the trypanosome status of tsetse flies (Tryp_negative and Tryp_positive). BRGp: *G. pallidipes* from Buffalo Ridge; BRGb: *G. brevipalpis* from Buffalo Ridge; ZuGp: *G. pallidipes* from Zungu Luka; ZuGa: *G. austeni* from Zungu Luka; MuGp: *G. pallidipes* from Mukinyo; MuGl: *G. longipennis* from Mukinyo; SaGl: *G. longipennis* from Sampu. (PDF 163 kb)

Additional file 5: **Figure S4.** Dimension 3 and 4 of the Multiple Correspondence Analysis 1 for explaining relationships between the trypanosome status of tsetse flies and their biological traits. (PDF 89 kb)

Additional file 6: **Table S2.** Adjusted Eta^2^ for the combination of variables in dimensions 1–3 in the Multiple Correspondence Analysis 1. (DOCX 13 kb)

Additional file 7: Summary of additional GLM analyses performed to consider: A) the tsetse-specific factors that affect presence or absence of *Sodalis glossinidius*, without taking into account trypanosome presence; B) the effects of site on *Sodalis glossinidius*, tested by considering only *G. pallidipes*; and C) the effects of site on trypanosome prevalence by focusing only on *G. pallidipes*. (DOCX 23 kb)

Additional file 8: **Figure S5.** The modified collection cage used with NG2G traps in this study. (PDF 2992 kb)

Additional file 9: **Table S3.** Primers sets used for screening parasites, vectors and endosymbionts. (DOCX 19 kb)

Additional file 10: **Table S4.** Comparison of the target location, amplification product size for the three PCR primer pairs tested (pSG2-Farikou, GPO1 and Hem) for the detection of *S. glossinidius* in tsetse flies. (DOCX 57 kb)

## Abbreviations

AAT: Animal African Trypanosomiasis
AB: Abdomen
BR: Buffalo Ridge
BRGb: *Glossina brevipalpis* from Buffalo Ridge
BRGp: *Glossina pallidipes* from Buffalo Ridge
Ga: *Glossina austeni*
Gb: *Glossina brevipalpis*
Gl: *Glossina longipennis*
GLM: Generalised Linear Models
Gp: *Glossina pallidipes*
HP: head plus proboscis
IAEA: International Atomic Energy Agency
ITS1: Internal transcribed spacer 1
LRT: Likelihood Ratio Test
MCA: Multiple Correspondence Analysis
Mu: Mukinyo
MuGl: *Glossina longipennis* from Mukinyo
MuGp: *Glossina pallidipes* from Mukinyo
PC: principal component
Sa: Sampu
SaGl: *Glossina longipennis* from Sampu
SIT: sterile insect technique
SHNR: Shimba Hills National Reserve
Tb: *T. brucei*
Tck: *T. congolense kilifi*
Tcs: *T. congolense savannah*
Tg: *T. godfreyi*
Ts: *T. simiae*
Tv: *T. vivax*
Zu: Zungu Luka
ZuGa: *Glossina austeni* from Zungu Luka
ZuGp: *Glossina pallidipes* from Zungu Luka
