# Supplementary material for: Sodalis glossinidius presence in wild tsetse is only associated with presence of trypanosomes in complex interactions with other tsetse-specific factors
Source: BMC Microbiol. 2018 Nov 23;18(Suppl 1):163. doi: 10.1186/s12866-018-1285-6 (PMC6251152; doi:10.1186/s12866-018-1285-6)
Supplement: Supplementary file 2 — Figure S1. Probability of T. congolense presence in tsetse samples from the best-fitting model (Model 3). pos_Sodalis: S. glossinidius-positive status. neg_Sodalis: S. glossinidius-negative status. This analysis shows the significant three-way interaction between sex, age and S. glossinidius status. (PDF 93 kb) [file 12866_2018_1285_MOESM2_ESM.pdf]

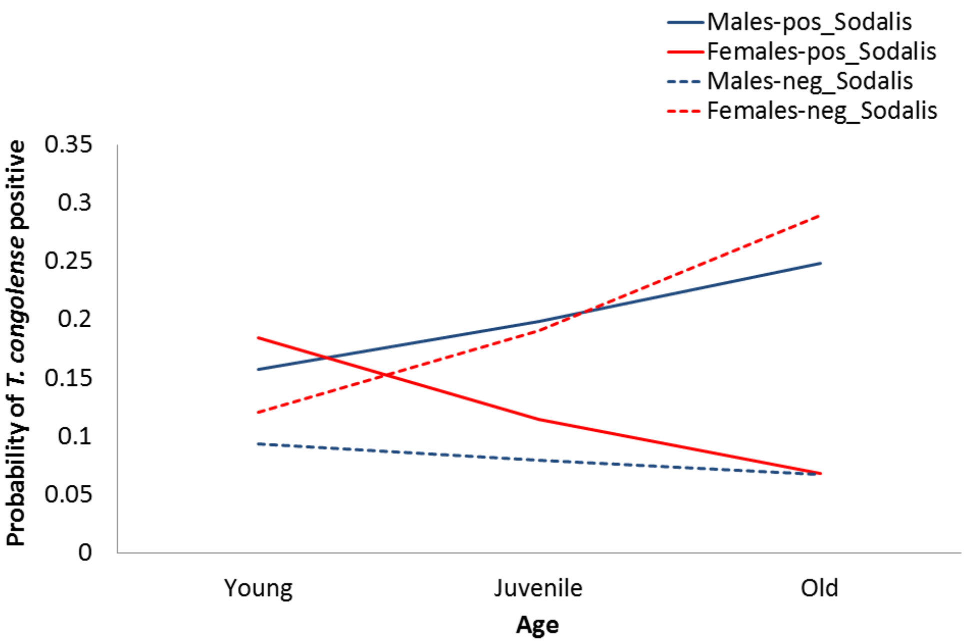

Figure S1. Probability of *T. congolense* presence in tsetse samples from the best-fitting model (Model 3). pos_Sodalis: *S.* glossinidius-positive status. neg_Sodalis: *S.* glossinidius-negative status. This analysis shows the significant three-way interaction between sex, age and *S. glossinidius* status.
