# Supplementary material for: Sodalis glossinidius presence in wild tsetse is only associated with presence of trypanosomes in complex interactions with other tsetse-specific factors
Source: BMC Microbiol. 2018 Nov 23;18(Suppl 1):163. doi: 10.1186/s12866-018-1285-6 (PMC6251152; doi:10.1186/s12866-018-1285-6)

**Figure S2: Probability of *T. brucei* presence in tsetse samples from the best-fitting model (Model 4), showing complex interactions.** Predicted values are shown for males and females in the seven subpopulations: (a) *G. pallidipes* from Buffalo Ridge. (b) *G. brevipalpis* from Buffalo Ridge. (c) *G. pallidipes* from Zungu Luka. (d) *G. austeni* from Zungu Luka. (e) *G. pallidipes* from Mukinyo. (f) *G. longipennis* from Mukinyo. (g) *G. longipennis* from Sampu.

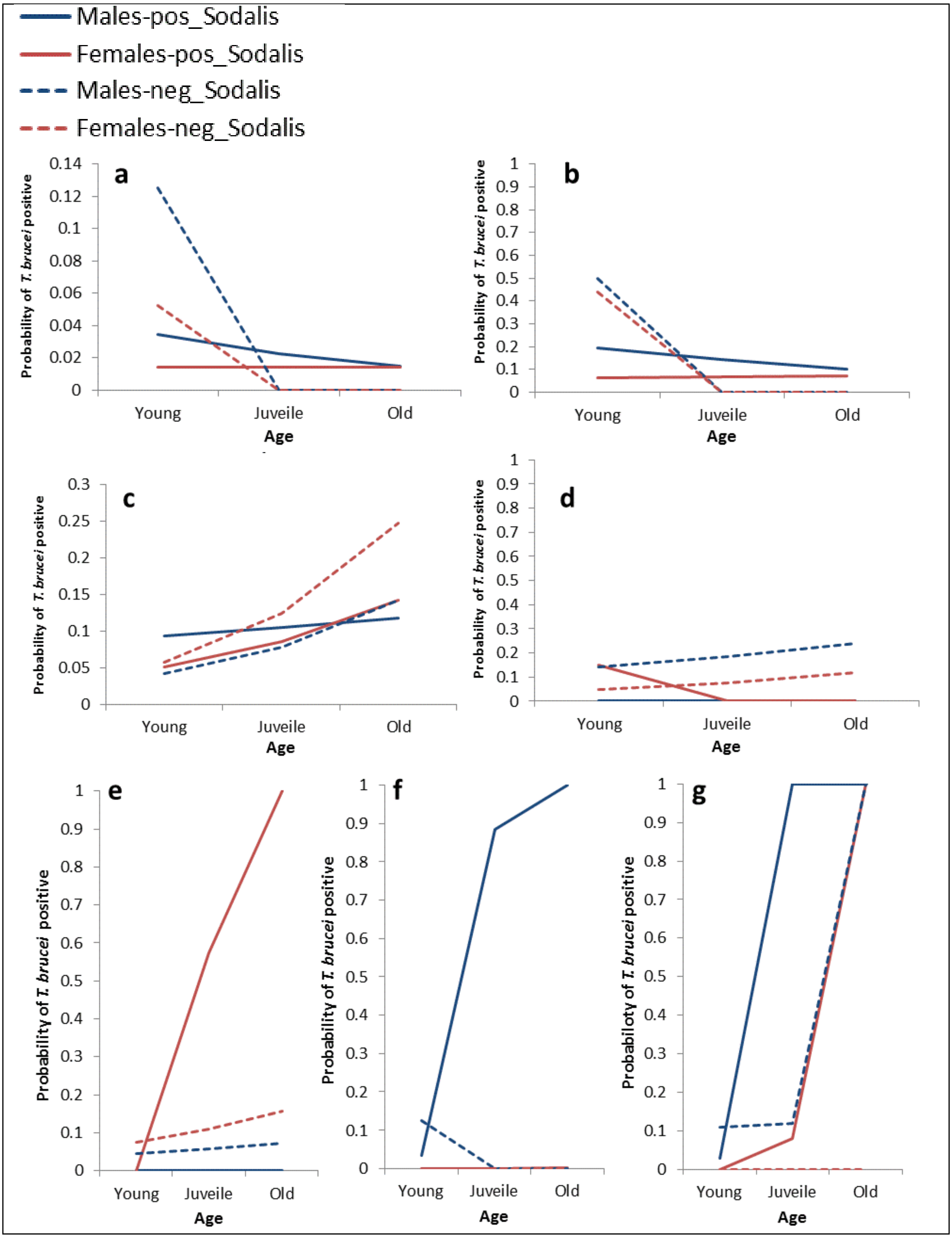

Supplement: Supplementary file 3 — Figure S2. Probability of T. brucei presence in tsetse samples from the best-fitting model (Model 4), showing complex interactions. Predicted values are shown for males and females in the seven subpopulations: (a) G. pallidipes from Buffalo Ridge. (b) G. brevipalpis from Buffalo Ridge. (c) G. pallidipes from Zungu Luka. (d) G. austeni from Zungu Luka. (e) G. pallidipes from Mukinyo. (f) G. longipennis from Mukinyo. (g) G. longipennis from Sampu. (PDF 140 kb) [file 12866_2018_1285_MOESM3_ESM.pdf]
