# Supplementary material for: Sodalis glossinidius presence in wild tsetse is only associated with presence of trypanosomes in complex interactions with other tsetse-specific factors
Source: BMC Microbiol. 2018 Nov 23;18(Suppl 1):163. doi: 10.1186/s12866-018-1285-6 (PMC6251152; doi:10.1186/s12866-018-1285-6)
Supplement: Supplementary file 4 — Figure S3. Dimensions 1 and 2 of the Multiple Correspondence Analysis 1 showing the relationships between the trypanosome status of tsetse flies and their biological traits. Sex (male and female), age (young, juvenile and old) and S. glossinidius status (negative and positive) of the seven subpopulations were plotted for describing associations with the trypanosome status of tsetse flies (Tryp_negative and Tryp_positive). BRGp: G. pallidipes from Buffalo Ridge; BRGb: G. brevipalpis from Buffalo Ridge; ZuGp: G. pallidipes from Zungu Luka; ZuGa: G. austeni from Zungu Luka; MuGp: G. pallidipes from Mukinyo; MuGl: G. longipennis from Mukinyo; SaGl: G. longipennis from Sampu. (PDF 163 kb) [file 12866_2018_1285_MOESM4_ESM.pdf]

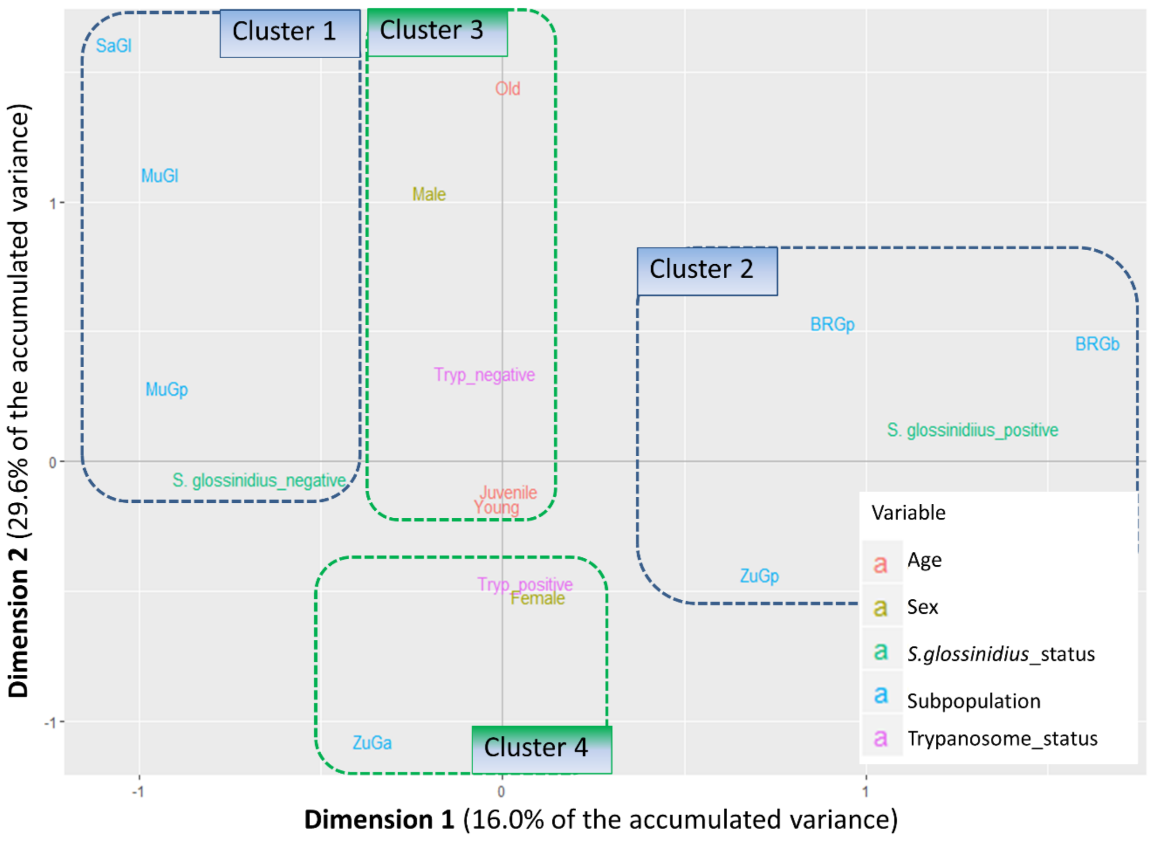

Figure S4. Dimensions 1 and 2 of the Multiple Correspondence Analysis 1 showing the relationships between the trypanosome status of tsetse flies and their biological traits. Sex (male and female), age (young, juvenile and old) and *S. glossinidius* status (negative and positive) of the seven subpopulations were plotted for describing associations with the trypanosome status of tsetse flies (Tryp_negative and Tryp_positive). BRGp: *G. pallidipes* from Buffalo Ridge; BRGb: *G. brevipalpis* from Buffalo Ridge; ZuGp: *G. pallidipes* from Zungu Luka; ZuGa: *G. austeni* from Zungu Luka; MuGp: *G. pallidipes* from Mukinyo; MuGl: *G. longipennis* from Mukinyo; SaGl: *G. longipennis* from Sampu.
