# Supplementary material for: Sodalis glossinidius presence in wild tsetse is only associated with presence of trypanosomes in complex interactions with other tsetse-specific factors
Source: BMC Microbiol. 2018 Nov 23;18(Suppl 1):163. doi: 10.1186/s12866-018-1285-6 (PMC6251152; doi:10.1186/s12866-018-1285-6)

Figure S4: Dimensions 3 and 4 of the Multiple Correspondence Analysis 1 for explaining relationships between the trypanosome status of tsetse flies and their biological traits.

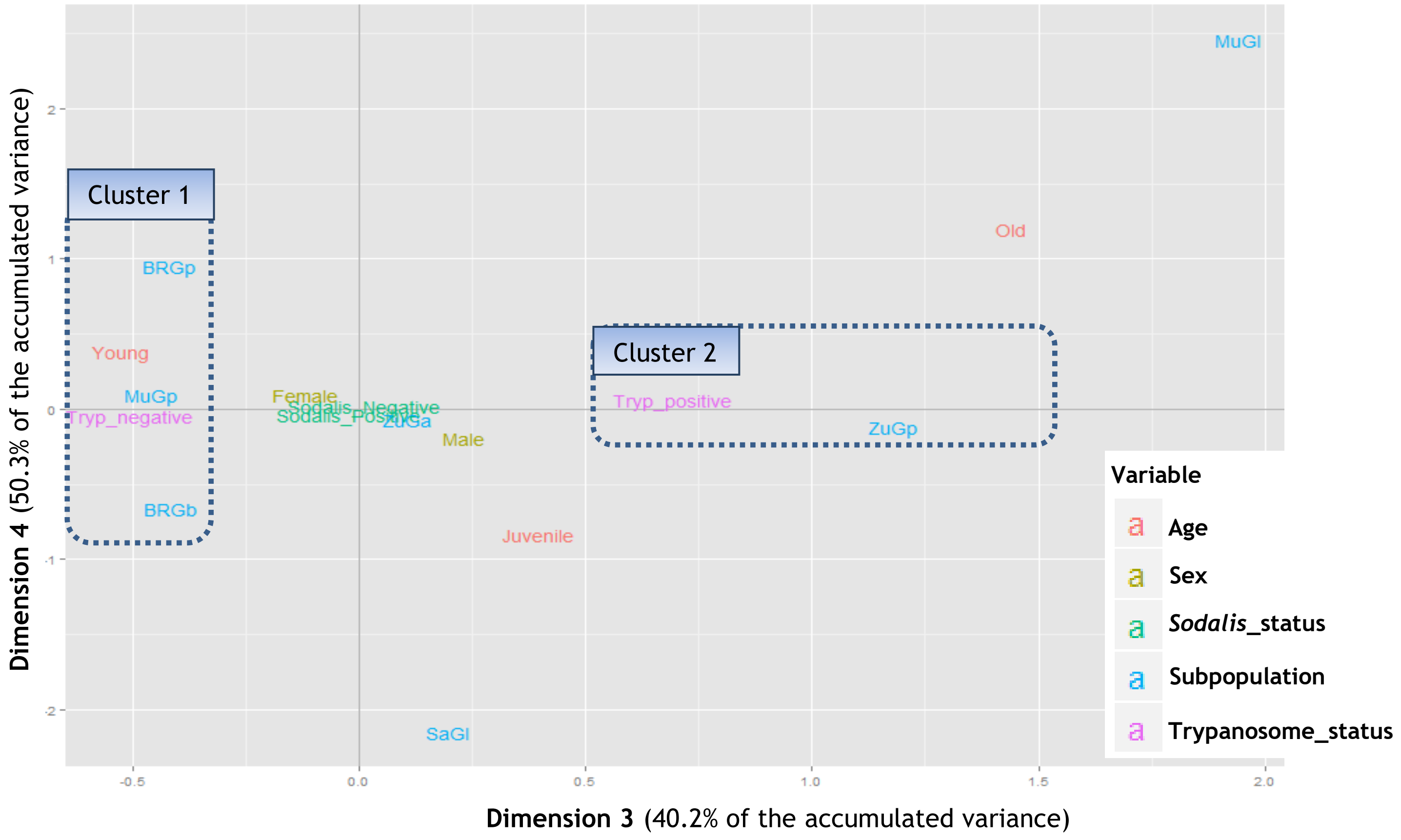

Supplement: Supplementary file 5 — Figure S4. Dimension 3 and 4 of the Multiple Correspondence Analysis 1 for explaining relationships between the trypanosome status of tsetse flies and their biological traits. (PDF 89 kb) [file 12866_2018_1285_MOESM5_ESM.pdf]
