# Supplementary material for: Sodalis glossinidius presence in wild tsetse is only associated with presence of trypanosomes in complex interactions with other tsetse-specific factors
Source: BMC Microbiol. 2018 Nov 23;18(Suppl 1):163. doi: 10.1186/s12866-018-1285-6 (PMC6251152; doi:10.1186/s12866-018-1285-6)
Supplement: Supplementary file 6 — Table S2. Adjusted Eta2 for the combination of variables in dimensions 1–3 in the Multiple Correspondence Analysis 1. (DOCX 13 kb) [file 12866_2018_1285_MOESM6_ESM.docx]

**Table S****2** Adjusted *Eta*^2^ for the combination of variables in dimensions 1-3 in the Multiple Correspondence Analysis 1.

| **Variables** | **Dimension 1** | **Dimension 2** | **Dimension 3** |
| --- | --- | --- | --- |
| *S. glossinidius*_status | 0.863 | 0.009 | 0.000 |
| subpopulation | 0.870 | 0.586 | 0.398 |
| sex | 0.020 | 0.537 | 0.025 |
| age | 0.000 | 0.210 | 0.385 |
| Trypanosome_status | 0.003 | 0.158 | 0.351 |
