# Supplementary material for: Sodalis glossinidius presence in wild tsetse is only associated with presence of trypanosomes in complex interactions with other tsetse-specific factors
Source: BMC Microbiol. 2018 Nov 23;18(Suppl 1):163. doi: 10.1186/s12866-018-1285-6 (PMC6251152; doi:10.1186/s12866-018-1285-6)
Supplement: Supplementary file 7 — Summary of additional GLM analyses performed to consider: A) the tsetse-specific factors that affect presence or absence of Sodalis glossinidius, without taking into account trypanosome presence; B) the effects of site on Sodalis glossinidius, tested by considering only G. pallidipes; and C) the effects of site on trypanosome prevalence by focusing only on G. pallidipes. (DOCX 23 kb) [file 12866_2018_1285_MOESM7_ESM.docx]

**Additional Analyses**

A) To consider the tsetse-specific factors that affect the presence or absence of *Sodalis glossinidius*, without taking into account trypanosomes, additional GLM analyses were conducted using *S. glossinidius* presence as a binary response variable and considering the fixed effects of subpopulation, sex, age and their interactions. The best-fitting model included only subpopulation. Posthoc tests (Tukey’s) indicated that there were significant differences between fly species both within and between sites, There were also significant differences for *G. pallidipes* at different sites, but only between the two regions sampled (i.e. BR and Zu at SHNR showing higher prevalence compared to Mu at Nguruman).

Fit: glm(formula = *S. glossinidius* ~ Subpopulation, family = binomial)

Linear Hypotheses:

| **Comparison** | **Difference** | **Std. Error** | **z value** | **Pr(>\|z\|)** |
| --- | --- | --- | --- | --- |
| BR_Gp - BR_Gb | -2.975 | 0.6096 | -4.881 | <0.001 *** |
| Mu_Gl - BR_Gb | -0.239 | 0.001153 | -0.019 | 1.000 |
| Mu_Gp - BR_Gb | -8.809 | 0.9187 | -9.588 | <0.001 *** |
| Zu_Ga - BR_Gb | -5.464e+00 | 6.054e-01 | -9.025 | <0.001 *** |
| Sa_Gl - BR_Gb | -2.239e+01 | 8.565e+02 | -0.026 | 1.000 |
| Zu_Gp - BR_Gb | -3.455e+00 | 6.103e-01 | -5.662 | <0.001 *** |
| Mu_Gl - BR_Gp | -1.942e+01 | 1.153e+03 | -0.017 | 1.000 |
| Mu_Gp - BR_Gp | -5.834e+00 | 7.311e-01 | -7.980 | <0.001 *** |
| Zu_Ga - BR_Gp | -2.489e+00 | 2.387e-01 | -10.426 | <0.001 *** |
| Sa_Gl - BR_Gp | -1.942e+01 | 8.565e+02 | -0.023 | 1.000 |
| Zu_Gp - BR_Gp | -4.800e-01 | 2.507e-01 | -1.915 | 0.367 |
| Mu_Gp - Mu_Gl | 1.359e+01 | 1.153e+03 | 0.012 | 1.000 |
| Zu_Ga - Mu_Gl | 1.693e+01 | 1.153e+03 | 0.015 | 1.000 |
| Sa_Gl - Mu_Gl | 1.054e-11 | 1.436e+03 | 0.000 | 1.000 |
| Zu_Gp - Mu_Gl | 1.894e+01 | 1.153e+03 | 0.016 | 1.000 |
| Zu_Ga - Mu_Gp | 3.345e+00 | 7.276e-01 | 4.597 | <0.001 *** |
| Sa_Gl - Mu_Gp | -1.359e+01 | 8.565e+02 | -0.016 | 1.000 |
| Zu_Gp - Mu_Gp | 5.354e+00 | 7.316e-01 | 7.317 | <0.001 *** |
| Sa_Gl - Zu_Ga | -1.693e+01 | 8.565e+02 | -0.020 | 1.000 |
| Zu_Gp - Zu_Ga | 2.009e+00 | 2.405e-01 | 8.353 | <0.001 *** |
| Zu_Gp - Sa_Gl | 1.894e+01 | 8.565e+02 | 0.022 | 1.000 |

---

Signif. codes:  0 ‘***’ 0.001 ‘**’ 0.01 ‘*’ 0.05 ‘.’ 0.1 ‘ ’ 1

B) To more specifically test the influence of site on *S. glossinidius* presence, GLM analyses were also conducted using only *G. pallidipes*. There was again only a significant effect of site, and the significantly higher prevalence of *S. glossinidius* at SHNR compared to Nguruman was confirmed.

Fit: glm(formula = *S. glossinidius* ~ Site, family = binomial)

Linear Hypotheses:

| **Comparison** | **Estimate** | **Std. Error** | **z value** | **Pr(>\|z\|)** |
| --- | --- | --- | --- | --- |
| Mu - BR | -5.8337 | 0.7310 | -7.981 | <0.001 *** |
| Zu - BR | -0.4800 | 0.2507 | -1.915 | 0.121 |
| Zu - Mu | 5.3537 | 0.7315 | 7.318 | <0.001 *** |

C) To separate the influence tsetse species from site on trypanosome presence, GLM analyses were conducted using only *G. pallidipes*. There was a significant interaction between site and sex but no significant effects of fly age or *S. glossinidius* presence. Due to the significant interaction, Tukey’s tests were conducted based on least squares ANOVA, after selecting the best-fitting model using likelihood ratio tests based on GLMS, under a binomial distribution. The output shows the confidence intervals. Comparisons in bold are significantly different. Except for BR (where there was a significant difference between females showed higher prevalence of trypanosomes than males), note that most significant differences were between Zu and the other sites (with higher prevalence at Zu) rather than within sites between sexes.

Fit: glm(formula = Tryps ~ Site + Sex + Site * Sex, family = binomial)

Interaction: Site:Sex

| **Comparison** | **Difference** | **Lower** | **Upper** | **p adjusted** |
| --- | --- | --- | --- | --- |
| Mu:Female-BR:Male | 0.03550112 | -0.147018943 | 0.2180212 | 0.9936810 |
| Mu:Male-BR:Male | 0.16101804 | -0.014887327 | 0.3369234 | 0.0946978 |
| **BR:Female-BR:Male** | **0.23349611** | **0.005359254** | **0.4616330** | **0.0413544** |
| **Zu:Male-BR:Male** | **0.33601804** | **0.139222362** | **0.5328137** | **0.0000201** |
| **Zu:Female-BR:Male** | **0.35991510** | **0.087476455** | **0.6323537** | **0.0024047** |
| Mu:Male-Mu:Female | 0.12551692 | -0.034883830 | 0.2859177 | 0.2222169 |
| BR:Female-Mu:Female | 0.19799499 | -0.018412330 | 0.4144023 | 0.0950011 |
| **Zu:Male-Mu:Female** | **0.30051692** | **0.117447966** | **0.4835859** | **0.0000493** |
| **Zu:Female-Mu:Female** | **0.32441398** | **0.061719255** | **0.5871087** | **0.0059378** |
| BR:Female-Mu:Male | 0.07247807 | -0.138380296 | 0.2833364 | 0.9233413 |
| Zu:Male-Mu:Male | 0.17500000 | -0.001474827 | 0.3514748 | 0.0534241 |
| Zu:Female-Mu:Male | 0.19889706 | -0.059245615 | 0.4570397 | 0.2378136 |
| Zu:Male-BR:Female | 0.10252193 | -0.126054297 | 0.3310982 | 0.7946039 |
| Zu:Female-BR:Female | 0.12641899 | -0.169792705 | 0.4226307 | 0.8268778 |
| Zu:Female-Zu:Male | 0.02389706 | -0.248909615 | 0.2967037 | 0.9998658 |
