# Supplementary material for: Sodalis glossinidius presence in wild tsetse is only associated with presence of trypanosomes in complex interactions with other tsetse-specific factors
Source: BMC Microbiol. 2018 Nov 23;18(Suppl 1):163. doi: 10.1186/s12866-018-1285-6 (PMC6251152; doi:10.1186/s12866-018-1285-6)

Figure S5. The modified collection cage used with NG2G traps in this study. The cage was made of a 1 L plastic water bottle at the top of the trap. The bottle contained 95% ethanol, was placed around 45° angle above the net and was fixed to the metallic structure of the trap with a string.

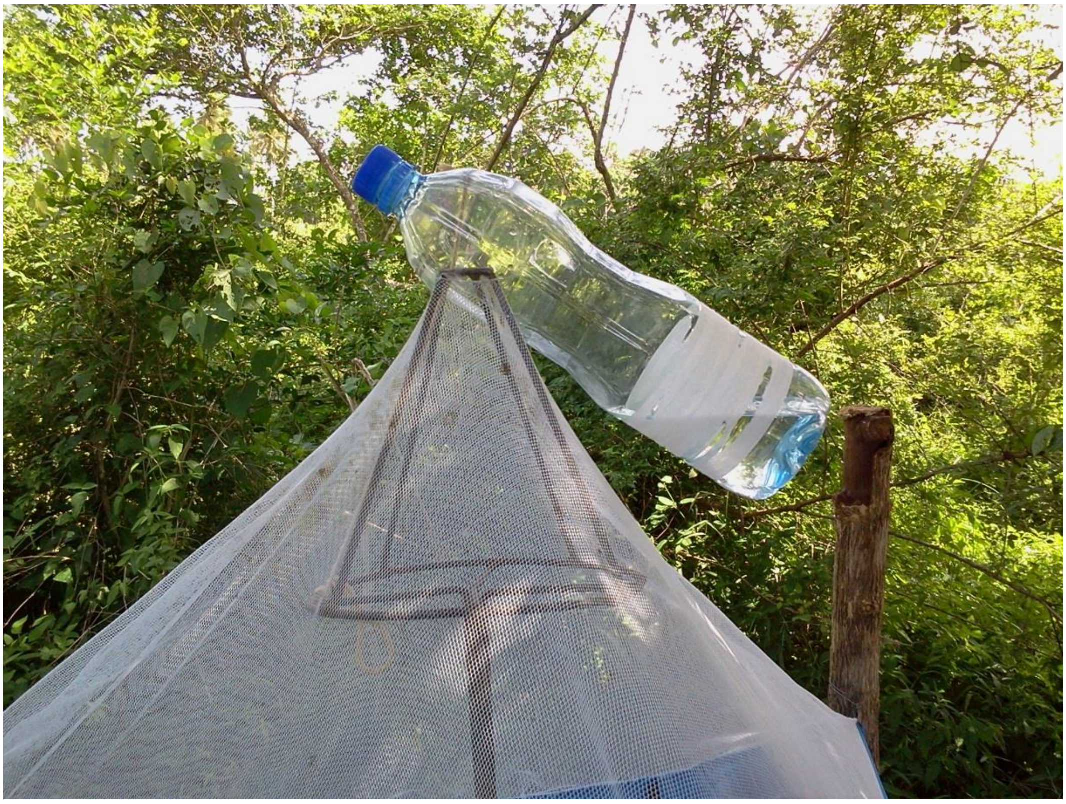

Supplement: Supplementary file 8 — Figure S5. The modified collection cage used with NG2G traps in this study. (PDF 2992 kb) [file 12866_2018_1285_MOESM8_ESM.pdf]
