# Supplementary material for: Sodalis glossinidius presence in wild tsetse is only associated with presence of trypanosomes in complex interactions with other tsetse-specific factors
Source: BMC Microbiol. 2018 Nov 23;18(Suppl 1):163. doi: 10.1186/s12866-018-1285-6 (PMC6251152; doi:10.1186/s12866-018-1285-6)
Supplement: Supplementary file 9 — Table S3. Primers sets used for screening parasites, vectors and endosymbionts. (DOCX 19 kb) [file 12866_2018_1285_MOESM9_ESM.docx]

**Table S3** Primers sets used for screening parasites, vectors and endosymbionts

| **Target** | **Amplicon size (bp)** | **Primer name** | **Primer sequence (5’-3’)** | **Ref.** |
| --- | --- | --- | --- | --- |
| *Trypanosoma* spp. ITS1 | 250 - 700^a^ | ITS1 CF | CCGGAAGTTACCGATATTG | [Njiru *et al.* 2005](#_ENREF_2) |
|  |  | ITS1 BR | TTGCTGCGTTCTTCAACGAA |  |
| *Glossina* spp. ITS1 | 778 - 1250^b^ | GlossinaITS1_for | GTGATCCACCGCTTAGAGTGA | [Dyer *et al.* 2008](#_ENREF_1) |
|  |  | GlossinaITS1_rev | GCAAAAGTTGACCGAACTTGA |  |
| *S. glossinidius* Hem | 650 | HemF | ATGGGAAACAAACCATTAGCCA | [Pais *et al.* 2008](#_ENREF_3) |
|  |  | HemR | TCAAGTGACAAACAGATAAATC |  |

^a^: Various sizes between 250 and 700 bp depending on the trypanosome(s) species present in the sample (*T.* *brucei* ssp.: ~480 bp; *T. congolense* savannah/forest:~700 bp, *T. congolense* kilifi ~620 bp; *T. simiae*: ~400 bp; *T. simiae* Tsavo: ~370 bp; *T. godfreyi*: ~300 bp and *T. vivax*: ~250 bp) . ^b^: Various sizes between 778 and 1250 bp depending on the *Glossina* species (see Dyer et al 2008 for details).

**References**

Dyer, N. A., S. P. Lawton, S. Ravel, K. S. Choi, M. J. Lehane *et al.*, 2008 Molecular phylogenetics of tsetse flies (Diptera: Glossinidae) based on mitochondrial (*COI*, *16S*, *ND2*) and nuclear ribosomal DNA sequences, with an emphasis on the palpalis group. Molecular Phylogenetics and Evolution 49**:** 227-239.

Njiru, Z. K., C. C. Constantine, S. Guya, J. Crowther, J. M. Kiragu *et al.*, 2005 The use of ITS1 rDNA PCR in detecting pathogenic African trypanosomes. Parasitology Research 95**:** 186-192.

Pais, R., C. Lohs, Y. N. Wu, J. W. Wang and S. Aksoy, 2008 The obligate mutualist *Wigglesworthia glossinidia* influences reproduction, digestion, and immunity processes of its host, the tsetse fly. Applied and Environmental Microbiology 74**:** 5965-5974.
