# Supplementary material for: Sodalis glossinidius presence in wild tsetse is only associated with presence of trypanosomes in complex interactions with other tsetse-specific factors
Source: BMC Microbiol. 2018 Nov 23;18(Suppl 1):163. doi: 10.1186/s12866-018-1285-6 (PMC6251152; doi:10.1186/s12866-018-1285-6)
Supplement: Supplementary file 10 — Table S4. Comparison of the target location, amplification product size for the three PCR primer pairs tested (pSG2-Farikou, GPO1 and Hem) for the detection of S. glossinidius in tsetse flies. (DOCX 57 kb) [file 12866_2018_1285_MOESM10_ESM.docx]

### Table S4: **Comparison** of the target location, amplification product size for the three PCR primer pairs tested (pSG2-Farikou, GPO1 and Hem) for the detection of *Sodalis glossinidius* in tsetse flies.

| **Primer pair** | **Location of the amplification** | **Fragment size (bp)** | **Reference** |
| --- | --- | --- | --- |
| pSG2-Farikou | 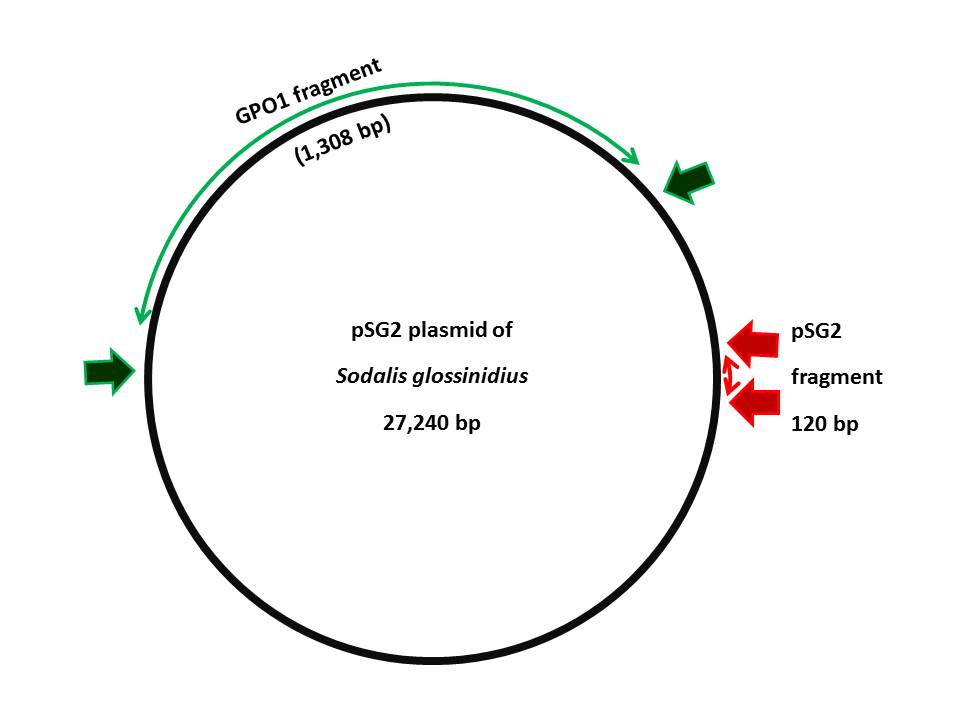 | 120 | ([Farikou *et al.* 2010](#_ENREF_1)) |
| GPO1 |  | 1,200 | ([O'Neill *et al.* 1993](#_ENREF_2)) |
| Hem | Nuclear hemolysin gene | 650 | ([Pais *et al.* 2008](#_ENREF_3)) |

Farikou, O., F. Njiokou, J. A. M. Mbida, G. R. Njitchouang, H. N. Djeunga *et al.*, 2010 Tripartite interactions between tsetse flies, *Sodalis glossinidius* and trypanosomes-An epidemiological approach in two historical human African trypanosomiasis foci in Cameroon. Infection Genetics and Evolution 10**:** 115-121.

O'Neill, S. L., R. H. Gooding and S. Aksoy, 1993 Phylogenetically distant symbiotic microorganisms reside in *Glossina* midgut and ovary tissues. Medical and veterinary entomology 7**:** 377-383.

Pais, R., C. Lohs, Y. N. Wu, J. W. Wang and S. Aksoy, 2008 The obligate mutualist *Wigglesworthia glossinidia* influences reproduction, digestion, and immunity processes of its host, the tsetse fly. Applied and Environmental Microbiology 74**:** 5965-5974.
